# Prospective, monocentric observational study on the clinical use and patient satisfaction of an implantable venous access Port

**DOI:** 10.1007/s00423-025-03654-3

**Published:** 2025-02-26

**Authors:** Alexander Werba, Roland Hennes, Fabian Schuh, Magdalena Holze, Barbara Maichle, Ali Ramouz, Mark Bauer, Kai Braunsteffer, Phillip Knebel, Rosa Klotz, Frank Pianka

**Affiliations:** 1 Department of General, Visceral and Transplantation Surgery, University of Heidelberg, Im Neuenheimer Feld 420, 69120 Heidelberg, Germany; 2 Study Center of the German Society of Surgery, University of Heidelberg, Heidelberg, Germany; 3 pfm Medical GmbH, Cologne, Germany

**Keywords:** Venous access ports, Patient satisfaction, TIVAP, Chemotherapy

## Abstract

**Purpose:**

Long-term totally implantable venous access ports (TIVAP) are essential for administering chemotherapy and parenteral nutrition in cancer patients. This study aimed to evaluate patient satisfaction and postoperative complications, compared to existing literature, emphasizing patient-reported outcomes (PRO) as a critical factor in treatment decisions.

**Methods:**

A prospective, monocentric observational study included 149 patients requiring TIVAP between April 13 and August 17, 2022. Patient satisfaction was assessed using a questionnaire covering four domains: overall satisfaction, willingness to choose the port again, cosmetic result, and pain experience. A meta-analysis of seven studies (1,035 patients) was performed to establish baseline data. The study assumed a non-inferiority margin of 12 points on a 0–100 scale (lower scores indicating better outcomes). Follow-ups were carried out at four weeks and six months.

**Results:**

Of the 149 patients (mean age 58.8 years, 96% with oncological diseases), 117 (78.5%) TIVAPs were implanted via the cut-down technique, primarily using the cephalic vein (84.6%). Out of 1,240 documented catheter accesses, 91.4% were complication-free. Satisfaction scores demonstrated non-inferiority across all domains compared to literature: overall satisfaction (18.5 vs. 35.6; *p* < 0.001), willingness to choose the port again (15.8 vs. 29.4; *p* < 0.001), cosmetic result (38.0 vs. 39.9; *p* = 0.003), and pain experience (17.1 vs. 31.5; *p* < 0.001).

**Conclusion:**

The study demonstrated adequate patient satisfaction and non-inferiority of the investigated TIVAP system compared to other systems, though no superiority was found in cosmetic outcomes.

**Supplementary Information:**

The online version contains supplementary material available at 10.1007/s00423-025-03654-3.

## Introduction

Worldwide, the burden of cancer is rising, with an estimated 19.3 million new cases and 10 million related deaths in 2020 [1]. Equivalently, by 2040, afflicted patients requiring chemotherapy are predicted to increase from 9.8 million to approximately 15 million annually [2]. The use of totally implantable venous access ports (TIVAP) has become the standard in clinical practice for the long-term administration of not only chemotherapeutic drugs, but also parenteral nutrition and antibiotics [3]. Either via closed cannulation, preferably of the subclavian or jugular vein, or an open cut-down technique into the cephalic vein a catheter is placed in the superior vena cava [4, 5]. Comparisons between both techniques and the related safety of the procedures have been conducted in the past with the open cut-down technique emerging as superior [5, 6].

Whereas procedural aspects have been highlighted previously, patient satisfaction has not been thoroughly evaluated prospectively [7, 8]. In addition to that, because of the rising understanding of the heterogeneity of tumors and the emerging individualized treatment options, patient involvement and satisfaction are of utmost importance [9]. Although there are multiple tools assessing the health-related quality of life (QoL), these questionnaires often lack the specificity for TIVAP-related patient satisfaction [10, 11]. In 2012, Nagel et al. designed a question cluster to assess patient-reported outcomes (PRO) regarding the above-mentioned parameters and the implanted TIVAP system [11]. Since then, only a few studies reported patient satisfaction after TIVAP implantation [11–14]. The implementation of PROs allows for a more accurate and focused assessment of crucial aspects of importance to the patient and is gaining increasing importance in clinical trials [15]. Additionally, PROs represented an independent predictor of overall survival in a large-scaled systematic review by Gotay et al. summarizing 13,874 patients, which further solidifies the crucial role of PROs.

This study aimed to evaluate patient satisfaction six months after TIVAP implantation using the above-mentioned questionnaire.

## Methods

This study was a prospective, monocentric, and single-arm observational study, initiated in collaboration with pfm medical as a post-market clinical follow-up study. The trial was approved by the local ethics committee (S-080/2022). In accordance with the Declaration of Helsinki [16] the trial was prospectively registered in an international and national database (NCT05209828, DRKS00026150) and reported according to the “Strengthening the Reporting of Observational Studies in Epidemiology (STROBE) Statement” [17].

Patients were included between April 13th and August 17th, 2022. The follow-up period after implantation of the TIVAP was six months. All procedures were conducted at the University Hospital Heidelberg. In addition to patient demographics and basic biometric data, the above-mentioned modified questionnaire (Online Resource 1) [11] was collected after six months and a patient diary was collected after the four-week and six-month follow-up. Patients with a minimum age of 18 years, an indication for a TIVAP implantation, and written informed consent were viable for inclusion. Exclusion criteria were defined as: contraindications for TIVAP implantation according to the manufacturer´s instruction for use, patients who already underwent implantation with TIVAP still in place, participants in other interventional trials that could interfere with the TIVAP or the primary endpoint of the presented study, patients who were unable to provide informed consent due to cognitive impairment or language barriers.

Outcome parameters, the related variables and the measurement method were assessed as follows: satisfaction was determined according to the patient questionnaire six months after implantation. Procedural aspects were defined as the method of implantation as well as the device used. Safety was defined as the complication rate including incidence and severity during and after implantation of the pfm medical TIVAP systems, measured by (serious) adverse events ((S)AEs) during the study period.

The primary endpoint concerning non-inferiority of patient satisfaction was based on four specific questions taken from the Satisfaction and QoL survey by Nagel et al. [11]:

- Overall, how satisfied are you with the port system? (Satisfied Overall)
- In a similar situation, would you choose the port again? (Choose Port Again)
- How satisfied are you with the cosmetic result? (Cosmetic Results)
- Does the port cause pain? (Pain)

Each question could be answered on a 5-step ordinal scale using the terms “very”, “quite”, “somewhat”, “a little” and “not at all”, which were converted to a numerical scale ranging between 0 (most positive answer) and 100 (most negative answer). These data were collected at the six-month follow-up.

Study success depended on success on all endpoints. An endpoint was considered successful if it was non-inferior to its reference mean value when considering a minimal clinically important difference (MCID) of 12 points. As the questionnaire does not allow for the calculation of a single total satisfaction score, the four domains were analyzed separately. To generate reliable baseline data for each question, a meta-analysis comprising eight studies (including the presented data) was performed [11–14, 18–20]. The analyzed studies were identified by a literature search via common databases such as pubmed, web of science, google scholar, including cross-referencing. Procedural aspects and complications were evaluated on-site as well as using the patient diaries.

### Surgical procedure

We adopted the open cut-down technique as our standard implantation method, as the PORTAS-3 trial demonstrated a significantly reduced incidence of pneumothorax or hemothorax (OR 0.27, *p* = 0.029) with this approach compared to the closed technique, while maintaining comparable success rates and tolerability [6]. The implantation side was determined contralaterally to the dominant hand if no other contraindications existed. After skin disinfection and patient placement in a slight Trendelenburg position, the skin incision in the infraclavicular fossa was made. Afterward, a sufficiently sized cephalic vein or similar vessel was exposed. In cases where no appropriate vessel was present a (modified) Seldinger´s technique was performed. After incision or puncture of the vein, the beforehand heparin-rinsed catheter was inserted in the vessel. Under fluoroscopy the tip of the catheter was placed in the superior vena cava. Subsequently, the subcutaneous pocket was prepared by blunt dissection, and the catheter was connected with the previously flushed chamber. The chamber was sutured to the underlying muscle fascia with a resorbable suture. The wound was closed starting with the subcutaneous layer followed by skin closure with a running resorbable suture. Functional control of the implanted system was judged by puncture of the chamber, aspiration of blood and infusion of saline solution. All patients received one of the following three medical devices: Jet Port^®^ Plus II Contrast, T-Port Contrast or T-Port Low Profile Contrast (pfm medical gmbh, Cologne). The TIVAP chamber is made of polyoxymethylene (POM) or titanium for Jet Port^®^ Plus II Contrast / T-Port Contrast or T-Port Low Profile Contrast, respectively. The septum is made of silicone and the catheter is made of polyurethane (PUR).

### Statistical analysis

The analysis of the differences in satisfaction between the patients and the reference values was performed using one-sample, one-sided t-tests with a significance level of 0.025. No imputation of data was performed. No multiplicity adjustment was necessary for the Type I error rate as the rejection of the null hypothesis requires all four individual domains to be successful for the primary endpoint to be successful. A general estimate of the MCID was considered half a standard deviation [21]. The Type II error rate was accounted for by using a Bonferroni correction method before sample size calculation [22]. In this framework, an overall power of 0.8 is achieved by setting the error rate for each question to 0.05. Using these parameters, the sample size calculation was carried out for each question individually, and the maximum value of 75 was then chosen. Assuming a drop-out rate of 50%, 150 patients were required to demonstrate a significant difference. The safety analysis was performed based on the intention to treat set (ITT), which consists of successfully treated patients as well as treatment failures. These patients received at least one safety assessment after enrolment. Descriptive statistics were based on the full analysis set (FAS), taking all patients into account that successfully received the study product. In addition to the FAS, the per-protocol set (PPS) was defined and used as the basis for testing the study hypothesis. The PPS is a subordinate of the FAS and comprised the “evaluable subjects” whose inclusion, treatment, and follow-up were conducted according to the clinical investigation plan, for whom the patient survey for the assessments of the key objectives could be performed, and for whom the data necessary for the statistical analysis of the study hypothesis is complete without missing values.

All statistical analyses were performed with R Version 1.4.1106 [23].

## Results

**Fig. 1:**
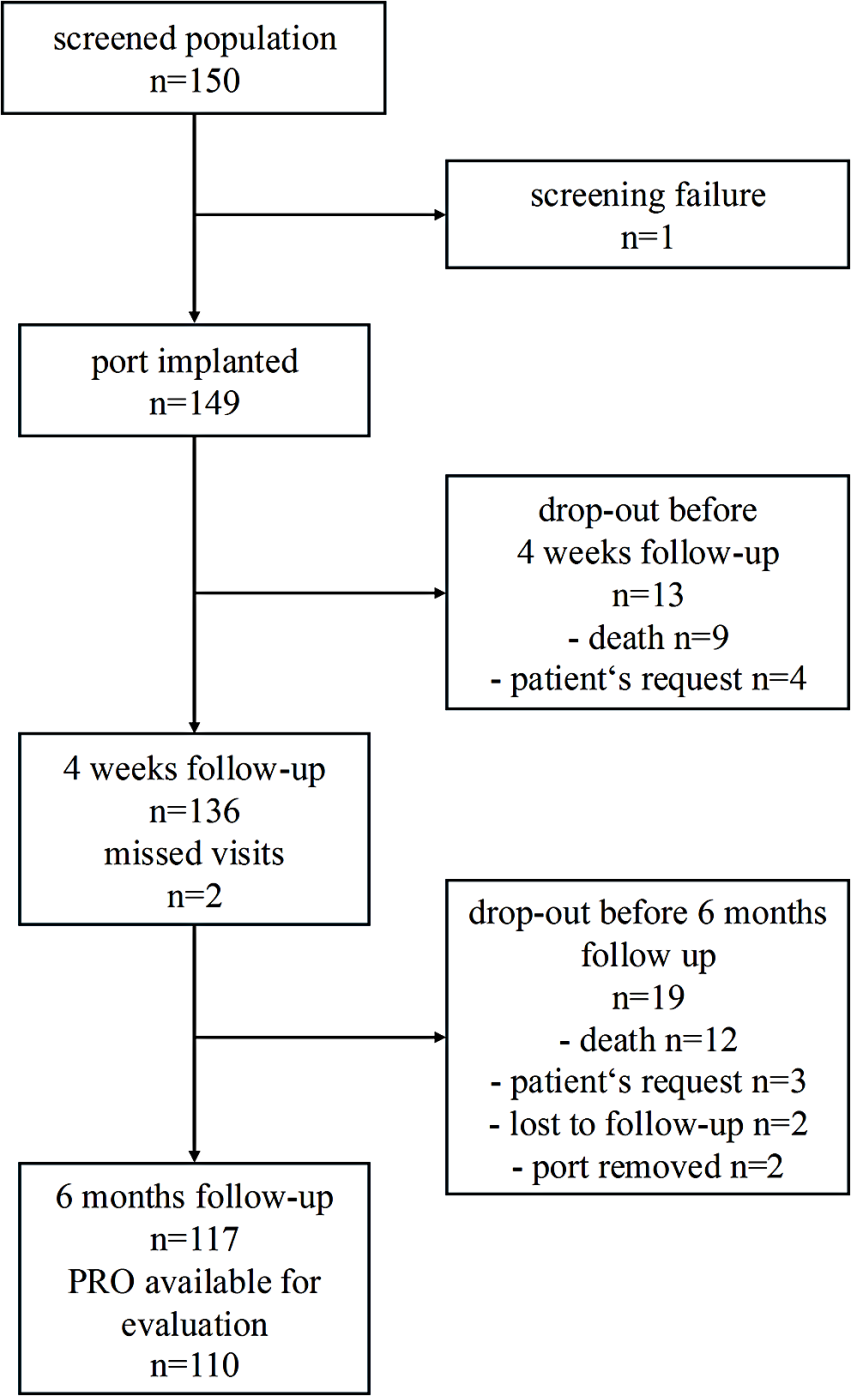
Flow diagram of included patients and exclusion criteria during the study period

Between April 13, 2022, and August 17, 2022, 149 patients (68 male (45.6%) and 81 female (54.4%) patients) with a mean age of 58.8 (± 13.0) years were included. A total of 110 patients reached the six-month follow-up and submitted survey data (Fig. 1). 137 (92%) of the TIVAPs were implanted by an expert-level board-certified surgeon. Overall, 117 patients completed the six months follow-up. 21 patients died before the study ended, seven patients withdrew their approval, two participants were lost to follow-up and in two cases the TIVAP system had to be removed because of infections. A total of 107 (71.8%) patients submitted at least one diary.

**Fig. 2:**
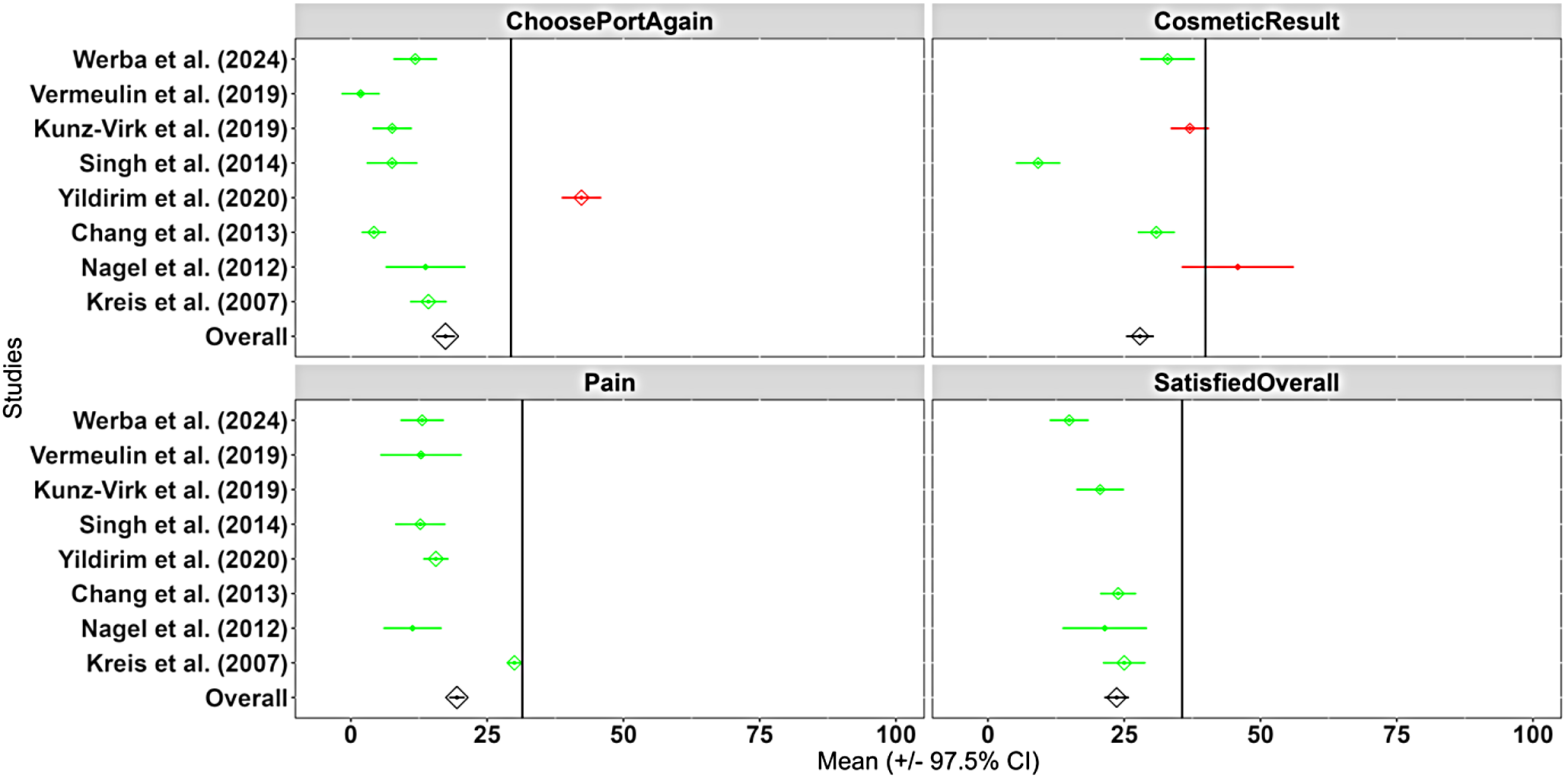
Forest plots for all four relevant domains and the corresponding score ranging from 0 (best) to 100 (worst)

The performed meta-analysis summarizes eight studies (including the presented study data) [11–14, 18–20], which include the questionnaire or the four domains of interest as described above. Overall, in the included studies, 1,035 patients completed the questionnaire and were therefore analyzed to generate baseline data for each question to identify mean values and standard deviations. Characteristics of the included studies can be seen in Table 1. The meta-analysis and corresponding forest plots are visualized in Fig. 2. The main indication for TIVAP placement was a planned chemotherapy. Six of seven studies used the Seldinger technique for TIVAP implantation [11–14, 18, 20]. Two studies did not declare the exact TIVAP system used [19, 20].

**Table 1:** Characteristics of included studies, all data as reported. Controlled clinical trial (CCT), chemotherapy (CTx), not specified (n.s.)

Author	Study date	Study design	*N* (m/f)	Implantation method	Main indication	Time of questionnaire	TIVAP system
Chang	2013	Observational	193 (122/71)	Seldinger technique	CTx	6 months	Bard PowerPort
Kreis	2007	Observational	232 (0/232)	Seldinger technique	CTx	10 months (± 5.03)	Baxter Port
Kunz-Virk	2019	Observational	1169 (520/549)	Seldinger technique	CTx	3 months	Bard PowerPort
Nagel	2012	Observational	98 (39/59)	Seldinger technique	CTx	6 months	Bard PowerPort
Singh	2014	CCT	114 (0/114)	Seldinger technique	CTx	197.5 days (± 83.9)	BardPort/ X-Port
Vermeulin	2019	Observational	57 (35/22)	n.s.	CTx	6 months (1–80)	n.s.
Yildirim	2020	Observational	263 (137/126)	Seldinger technique	CTx	Min. 4 weeks	n.s.

For more than 95% of the included patients, the indication for TIVAP implantation was a planned or ongoing chemotherapy. Moreover, approximately 30% of all patients needed TIVAP implantation for recurrent blood collection. The baseline characteristics of the study population can be seen in Table 2.

**Table 2:** Baseline characteristics of study population

	*N* (%) or mean (SD)
Sex Male Female	68 (45.6%) 81 (54.4%)
Age (years)	58.8 (13.0)
Body weight (kg)	75.5 (17.4)
Height (cm)	170.9 (9.3)
Body mass index (kg / m^2^)	25.7 (5.0)
Karnofsky Index 100% 90% 80% 70% 60% 50% < 50% Missing	50 (33.6%) 32 (21.5%) 19 (12.8%) 15 (10.1%) 17 (11.4%) 10 (6.7%) 3 (2.0%) 3 (2.0%)
Handedness Right Left Missing	135 (90.6%) 12 (8.1%) 2 (1.3%)
Smoking Status Nonsmoker Active smoker Previous smoker	71 (47.7%) 17 (11.4%) 61 (40.9%)
Alcohol consumption No Occasionally Regularly	50 (33.6%) 92 (61.7%) 7 (4.7%)
Immunosuppressive therapy No Yes	120 (80.5%) 29 (19.5%)
Active Antibiotic therapy No Yes Missing	135 (90.6%) 12 (8.1%) 2 (1.3%)
Active Chemotherapy No Yes	115 (77.2%) 34 (22.8%)
Diabetes mellitus No Yes	132 (88.6%) 17 (11.4%)
Planned Chemotherapy Performed Chemotherapy	143 (96.0%) 100 (67.1%)
Planned repeated blood collection Performed blood collection	47 (31.5%) 56 (37.6%)

117 (78.5%) of TIVAPs were successfully implanted using the open cut-down technique. In 27 (18.1%) cases a modified Seldinger approach was used to establish venous access. The main vessel used for catheter insertion was the cephalic vein (84.6%). Two TIVAPs were implanted in the femoral vein. Almost all interventions were performed under local anesthesia and only two (1.3%) under analgosedation. All but one catheter position was controlled via fluoroscopy, the latter via ultrasound. 146 of 149 patients (98%) were treated in an outpatient setting. Table 3 summarizes the procedural aspects of TIVAP implantation.

**Table 3:** Procedural aspects of TIVAP implantation

	*N* (%) or mean (SD)
Implantation method cut-down technique Seldinger technique modified Seldinger technique	117 (78.5%) 5 (3.4%) 27 (18.1%)
Implantation vein cephalic non-specified collateral subclavian internal jugular external jugular femoral	126 (84.6%) 13 (8.7%) 4 (2.7%) 2 (1.3%) 2 (1.3%) 2 (1.3%)
TIVAP fixation 2-point (connector + port hole) 3-point (connector + 2 port holes)	141 (94.6%) 8 (5.4%)
Implanted TIVAP and catheter size Jet Port^®^ Plus II Contrast 6.6 F T-Port Contrast 6.6 F T-Port Low Profile Contrast 6.6 F	81 (54.4%) 62 (41.6%) 6 (4.0%)
Implantation side left right	101 (67.8%) 48 (32.2%)
Operation time (minutes)	15.1 (10.7)
Intervention setting outpatient inpatient	146 (98.0%) 3 (2.0%)
Radiation dose (mGy x cm^2^)	325.3 (349.4)
Previous central venous catheter	41 (27.5%)

Overall, during the study period, access to a TIVAP was recorded 1,240 times with 746 (60.2%) of punctures succeeding the first time. After use, the TIVAP system was most commonly blocked with sodium chloride. A detailed listing of all rinsing agents used can be seen in Online Resource 2.

Concerning the primary study hypothesis, patient satisfaction was non-inferior to the reference value of the comparison literature in all four endpoint-related domains from the questionnaire: Overall satisfaction (18.5 vs. 35.6; *p* < 0.001), the willingness to choose the port again (15.8 vs. 29.4; *p* < 0.001), the cosmetic result (38.0 vs. 39.9; *p* = 0.003), and whether the port was painful for the patient (17.1 vs. 31.5; *p* < 0.001). An overview of the results of the main outcome can be seen in Table 4.

**Table 4:** Overview of the results of the main outcome. CI = confidence interval, MCID = minimal clinically important difference

Domain	*N*	Mean	Mean reference studies	*p*-value
Satisfied Overall	109	14.9 ± 19.0 (upper 97.5%-CI = 18.5)	23.6 (+ 12 (MCID) = 35.6)	< 0.001
Choose Port Again	108	11.8 ± 21.2 (upper 97.5%-CI = 15.8)	17.4 (+ 12 (MCID) = 29.4)	< 0.001
Cosmetic Result	110	33.0 ± 26.9 (upper 97.5%-CI = 38.0)	27.9 (+ 12 (MCID) = 39.9)	= 0.003
Pain	109	13.1 ± 21.1 (upper 97.5%-CI = 17.1)	19.5 (+ 12 (MCID) = 31.5)	< 0.001

It was further tested whether patient satisfaction was also superior to the reference literature, but due to the non-significant cosmetic result, no such statement could be concluded.

Furthermore, we conducted subgroup analyses comparing sex and age in reference to the four domains. Statistically significant results could not be observed at a significance level of 0.05 due to corrected p-values that are necessary to account for these additionally performed tests. Pain perception showed a mean difference between the age groups < 60 and ≥ 60 years (16.8 vs. 9.3; *p* = 0.4). The age split was based on the median age value of 59.5 years for patients that submitted the questionnaire.

Similarly, there was an observable mean difference between pain perception for female and male patients (16.7 vs. 8.2; *p* = 0.461).

During this study, in addition to standard procedural recording of complications, the patients were able to record emergent complications independently in a diary and report them at the subsequent follow-up visits. In the collected patient diaries, 41entries were related to problems after TIVAP implantation, with most of the entries describing hematomas (*n* = 14). Other patient-reported problems were occlusion without the need for removal of the TIVAP (*n* = 1) and infection (*n* = 4). Until and including the six-month FU, a total number of 21 (S)AE events in 21 patients (14.09% of treated patients) were received and evaluated (see Table 5). For one of those events, additional information (e.g., worsening, new treatment) was provided in a follow-up report. Initial reports and their respective follow-up reports were treated as a single (S)AE event.

**Table 5:** Overview of adverse events and categorization, (serious) adverse event ((S)AE)

Type of (S)AE	AE	SAE	Total
Bleeding	1	0	1 (4.8%)
Cardiac arrhythmia	0	1	1 (4.8%)
Hematoma	1	1	2 (9.5%)
Infection	2	6	8 (38.1%)
Thrombosis	4	0	4 (19%)
Wound healing disorder	1	0	1 (4.8%)
Wound infection	2	0	2 (9.5%)
Syncope	2	0	2 (9.5%)
Total	13	8	21 (100.0%)

## Discussion

This study aimed to investigate PROs after TIVAP implantation and was included in a pooled -meta-analysis of eight studies. Participants reported adequate satisfaction in the four relevant domains ‘satisfied overall’, ‘choose port again’, ‘cosmetic result’, and ‘pain’. This allows conclusions to be drawn about the quality of life of this patient cohort due to the known positive correlation between quality of life and patient satisfaction [24]. The findings of this study show non-inferiority of the implanted TIVAP system in comparison to the comparable literature [11–14, 18–20]. As a statistically significant superior cosmetic outcome could not be shown, we believe this to be most likely due to the highly standardized nature of TIVAP implantation, as there is minimal to no possible improvement expected to be made concerning wound closure and thus the cosmetic result.

In the original study by Nagel et al. [11] similar results were observed. In addition to that, the cosmetic outcome remains subjective, lacking an objective method to measure adequately. Besides these factors, patient-reported outcomes are influenced by a range of factors including demographics, health status, disease type, and the resulting prognosis [25]. Overall, patients receiving chemotherapy report worse QoL in comparison to other illnesses [26]. In our collective more than 95% of patients had TIVAP implantation due to a planned chemotherapy due to oncological diseases. Therefore, the results of this study mainly describe oncological patients’ satisfaction and should be interpreted as such.

Despite the focus of describing PROs after TIVAP implantation certain procedural and postinterventional aspects can be highlighted. In our institution, the standard implantation method was the open cut-down technique, as described above. Six out of the seven of the included studies primarily used the Seldinger technique for TIVAP implantation [11–14, 18, 20]. In our study, a total of 14.09% of patients experienced some form of complication, similar to comparable studies [13, 18, 20]. A large randomized controlled trial, including 1205 patients, compared an open versus a closed approach for TIVAP implantation and found no significant differences in tolerability, overall morbidity or primary implantation success rate [6]. Given these findings, we do not expect the choice of implantation method to have a substantial impact on PROs. Furthermore, TIVAP implantation is both feasible and safe in an outpatient setting. Additionally, data suggests longer infection-free survival in an ambulatory setting [27, 28]. However, patients with significant comorbidities or those requiring further concurrent surgical procedures may necessitate inpatient admission. In the presented study, 98% of TIVAP placements were conducted in an outpatient setting, following the local standard operating procedure.

According to the Food and Drug Administration (FDA), PROs are defined as follows: A PRO is any report of the status of a patient’s health condition that comes directly from the patient, without interpretation of the patient’s response by a clinician or anyone else’ [29]. Overall, 107 patients returned the diary after the six-month follow-up summarizing 1,240 entries. In approximately 60% of cases, patients reported that only one attempt was necessary to access the TIVAP via puncture, which likely contributed positively to three out of the four investigated domains. For more than 95% of patients TIVAP implantation was conducted due to a planned chemotherapy. Although only 100 (67.1%) patients received chemotherapy, the TIVAPs were later used for administration of medication (50, 33.6%), blood sampling (56, 37.6%), and the admittance of blood transfusions (16, 10.7%). This further reinforces the role of TIVAPs in oncological patients.

In recent years the patients’ perspective regarding health conditions related to the treatment gained well-deserved importance in comparison to other more commonly used surrogate parameters [30]. There are multiple factors influencing the perception of the importance and severity of a symptom, especially when assessing PROs [31]. This study evaluated patients undergoing TIVAP implantation at certain time points, which may lead to a detection bias of certain symptoms. Therefore, the implementation of PROs leads to a more detailed and accurate insight into the complexity of certain complications with a more realistic presentation of the patient’s treatment pathway and the acquired data.

To demonstrate the safety and performance according to the Medical Device Regulation (MDR), this study was designed as a post-market clinical follow-up (PMCF) study on the pfm medical vascular access ports Jet Port^®^ Plus II Contrast, T-Port Contrast, and T-Port Low Profile Contrast. Nevertheless, these TIVAP systems were already in everyday use in our institution mitigating the arising bias due to the sponsoring.

Certain limitations apply to this study. The reference literature was mainly based on a collective of patients summarized in the above-mentioned meta-analysis. Although studies, viable for inclusion, were adequately selected this leads to a certain bias. In addition to that, this study used only certain types of TIVAPs, which were not used in the referenced studies. Although our study center already used these products beforehand, this may lead to a distortion of the presented data.

## Conclusion

Patients undergoing implantation of TIVAPs report adequate postoperative satisfaction in terms of overall satisfaction, choosing the port again, cosmetic results and pain in comparison to previous studies. The findings of this study further underscore the critical role of PROs in understanding the impact of TIVAP implantation on patients’ lives.

## Electronic supplementary material

Below is the link to the electronic supplementary material.

Supplementary Material 1

Supplementary Material 2

## Data Availability

No datasets were generated or analysed during the current study.
