# Supplementary material for: Prospective, monocentric observational study on the clinical use and patient satisfaction of an implantable venous access Port
Source: Langenbecks Arch Surg. 2025 Feb 26;410(1):84. doi: 10.1007/s00423-025-03654-3 (PMC11865111; doi:10.1007/s00423-025-03654-3)
Supplement: Supplementary file 1 — Supplementary Material 1 [file 423_2025_3654_MOESM1_ESM.pdf]

Ausfülldatum: Patienten-ID:

Patientenfragebogen zum Port

Bitte lesen Sie jede Frage und kreuzen Sie dann die Antwort an, die Ihre Situation am besten beschreibt. Wenn Sie sich bei der Beantwortung einer Frage nicht sicher sind, wählen Sie die Antwort, die dem, wie Sie sich fühlen, am nächsten kommt. Bitte beantworten Sie alle Fragen.

	sehr	ziemlich	etwas	ein wenig	überhaupt nicht	
**1. Wie zufrieden sind Sie insgesamt mit dem Portsystem?**	1	2	3	4	5	
**2. Würden Sie sich in einer ähnlichen Situation wieder für einen Port entscheiden?**	1	2	3	4	5	
**3. Haben Sie Angst vor Komplikationen mit dem Port?**	1	2	3	4	5	
**4. Denken Sie öfter an den Port?**	1	2	3	4	5	
**5. Empfinden Sie den Port als störenden Fremdkörper?**	1	2	3	4	5	
**6. Wie zufrieden sind Sie mit dem kosmetischen Ergebnis der Implantation?**	1	2	3	4	5	
**7. Sind Sie ausreichend über den Operationsablauf zur Implantation aufgeklärt worden?**	1	2	3	4	5	
**8. War die Implantation schmerzhaft?**	1	2	3	4	5	
**9. Verursacht der Port ansonsten Schmerzen?**	1	2	3	4	5	
**10. Haben Sie Angst vor dem Anstechen der Portkammer?**	1	2	3	4	5	
**11. Sind Sie der Meinung, dass Ihre Behandlung durch die Implantation des Portsystems vereinfacht wurde?**	1	2	3	4	5	
**12. Behindert Sie das Portsystem beim Ausüben Ihrer täglichen Aktivitäten?**	1	2	3	4	5	
**13. Können Sie mit dem Port Ihre Freizeit genießen?**	1	2	3	4	5	
**14. Einen Port zu haben hat sich positiv auf meine Lebensqualität ausgewirkt.**	1	2	3	4	5	
	**sehr**	**ziemlich**	**etwas**	**ein wenig**	**überhaupt nicht**	**trifft auf mich nicht zu**
**15. Haben Sie das Gefühl, dass Sie bei der Arbeit durch den Port behindert werden?**	1	2	3	4	5	6
**16. Haben Sie das Gefühl, dass Sie beim Sport durch den Port behindert werden?**	1	2	3	4	5	6

*Nach dem Fragebogen von Nagel *et al*., 2011 und Yildirim *et a*l., 2020

Revision 1.0, 26.07.2021
