# Supplementary material for: Prospective, monocentric observational study on the clinical use and patient satisfaction of an implantable venous access Port
Source: Langenbecks Arch Surg. 2025 Feb 26;410(1):84. doi: 10.1007/s00423-025-03654-3 (PMC11865111; doi:10.1007/s00423-025-03654-3)
Supplement: Supplementary file 2 — Supplementary Material 2 [file 423_2025_3654_MOESM2_ESM.docx]

|  | N (%) |
| --- | --- |
| Sodium Chloride 0.9% | 474 (38.2%) |
| Taurolidine | 139 (11.2%) |
| Heparin | 18 (1.5%) |
| Unknown rinsing agent | 189 (15.2%) |
| No rinsing agent used | 420 (33.9%) |

**Online Resource 2** Rinsing agents used, according to patient diary
